# Cerebral venous hemodynamic disturbances in children with different types of headache. The role of transcranial ultrasonography methods

**DOI:** 10.1186/1129-2377-14-S1-P10

**Published:** 2013-02-21

**Authors:** MF Abramova, IA Stepanova, SN Novoselova

**Affiliations:** 1 Russian National Research, Medical University Named After N.I. Pirogov, Russian Federation

## Materials and methods

1,340 patients aged from 3 to 17 years who complained of headache have been examined. Ultrasonic Transcranial Doppler (TCD) of “BIOSS”and“SPECTROMED” companies (Russia),t ranscranial color-coded duplex (TCCD) by “Logic P-5”.

## Results

All children with headaches were separated according the clinical and ultrasound findings: migraine, tension type of headache, headache with increase or reduction of arterial pressure, headache caused by cerebral venous dysfunction. 30% of them had cerebral anomalies (of craniovertebral junction and deep brain veins). The children complained of headaches (100%) and also vegetative dysfunction (80%), nasal bleeding (60%), vomiting (40%), dizziness and noise in ears (35%). Venous outflow in the cavernous sinus, straight sinus, great cerebral vein of Galen have been registered by TCD, TCCD. Cerebral venous hemodynamic disturbances (“markers”) revealed in all groups children: migraine – 35 %, tension type of headache – 40 %, headache with increase or reduction of arterial pressure – from 25 % to 55 %. Thus a venous outflow is stimulated and makes influence on intracranial venous circulation. The estimation of cerebral venous hemodynamic disturbances in literature is described mainly in adults, but they are of great importance in clinical manifestations, especially in children. Diagnosis of such disturbances in children is not detected in time, though they often turn out to be one of the main evidence of cerebrovascular pathology. The complex research of cerebral venous hemodynamics presents new possibilities for revealing disturbances of cerebral venous blood circulation. The conservative treatment which has been performed under ultrasonographic control (TCD, TCCD) in children with disturbances of cerebral hemodynamics, led to objective improvement in 85% of children.

